# Epigallocatechin Gallate Reduces Ischemia/Reperfusion Injury in Isolated Perfused Rabbit Hearts

**DOI:** 10.3390/ijms19020628

**Published:** 2018-02-23

**Authors:** Aida Salameh, Roxana Schuster, Ingo Dähnert, Johannes Seeger, Stefan Dhein

**Affiliations:** 1 Heart Centre Clinic for Paediatric Cardiology, University of Leipzig, 04289 Leipzig, Germany; roxanaschuster@gmx.de (R.S.); ingo.daehnert@medizin.uni-leipzig.de (I.D.); 2 Institute of Veterinary Anatomy, Histology and Embryology, University of Leipzig, 04103 Leipzig, Germany; seeger@vetmed.uni-leipzig.de; 3 Rudolf-Boehm-Institute for Pharmacology and Toxicology, University of Leipzig, 04107 Leipzig, Germany; stefan.dhein@medizin.uni-leipzig.de

**Keywords:** EGCG, cardioplegia, ischemia/reperfusion injury, Langendorff

## Abstract

Cardioplegic arrest during heart operations is often used in cardiac surgery. During cardioplegia, the heart is subjected to a global ischemia/reperfusion-injury. (−)-epigallocatechin gallate (EGCG), one of the main ingredients of green tea, seems to be beneficial in various cardiac diseases. Therefore, the aim of our study was to evaluate EGCG in a rabbit model of cardioplegic arrest. Twenty four mature Chinchilla rabbits were examined. Rabbit hearts were isolated and perfused according to Langendorff. After induction of cardioplegia (without and with 20 µmol/L EGCG, *n* = 6 each) the hearts maintained arrested for 90-min. Thereafter, the hearts were re-perfused for 60 min. During the entire experiment hemodynamic and functional data were assessed. At the end of each experiment, left ventricular samples were processed for ATP measurements and for histological analysis. Directly after cessation of cardioplegia, all hearts showed the same decline in systolic and diastolic function. However, hearts of the EGCG-group showed a significantly faster and better hemodynamic recovery during reperfusion. In addition, tissue ATP-levels were significantly higher in the EGCG-treated hearts. Histological analysis revealed that markers of nitrosative and oxidative stress were significantly lower in the EGCG group. Thus, addition of EGCG significantly protected the cardiac muscle from ischemia/reperfusion injury.

## 1. Introduction

For thousands of years, tea has been one of the most popular beverages in Asian countries. Especially, green tea produced from leafs of the tea plant Camellia sinensis appears to be highly appreciated not only as a food product but also as a remedy for various diseases [1]. Green tea contains considerable amounts of phytochemicals such as flavonoids and polyphenols of which the catechins, and more precisely (−)-epigallocatechin gallate (EGCG), are among the main ingredients [2].

It has been proposed that regular consumption of green tea is associated with a lower risk of cancer and cardiovascular diseases [3]. Several studies in humans have been published, describing a reduced mortality and less arrhythmia after myocardial infarction in patients who are habitual tea drinkers [4,5]. Other studies revealed comparable results: the risk of dying due to cardiovascular events was lower in the patient group with regular intake of flavonoids (green tea or other sources) [6,7,8]. Additionally, several animal in vitro and in vivo investigations also demonstrated reduced infarct sizes, less apoptosis and a better outcome in the EGCG treatment groups [9,10,11]. Thus, EGCG seems to exert protective effects on the cardiac muscle during local ischemia [9,12] and global ischemia [13]. The cardioprotective effects of EGCG in these models of cardiac ischemia encouraged us to test whether EGCG may also exert cardioprotective effects when added to a cardioplegic solution during a 90-min cardiac arrest.

In cardiac operations, the whole heart is arrested, using cardioplegic solutions like Custodiol^®^, and thus, exposed to global ischemia. During cardioplegia, systemic circulation is maintained with a heart-lung machine and depending on the heart operation carried out, cardioplegia is continued for up to 90 min or even longer. Under these conditions, although the cardioplegic solutions are somehow cardio-protective, the heart is subjected to a severe ischemia/reperfusion injury, which often results in a decrease of right and left ventricular function impairing patient outcomes [14]. Under these conditions, especially during the reperfusion phase, reactive oxygen radicals arise, which damage cell membranes and are at least partially responsible for the observed tissue damage [15]. Therefore, appropriate treatments must be found to better protect the heart during arrest. One protective agent might be EGCG with its radical scavenging activity. In an animal study on diabetic rats undergoing cardiopulmonary bypass without or with EGCG supplementation, Funamoto et al. found that EGCG pre-treatment significantly reduced tubular damage in the kidney [16]. Additionally, our working group demonstrated, in an animal model of CPB, that EGCG was effective to protect kidneys, hippocampus and lung tissue of small piglets from CPB-associated injury [17,18,19]. Thus, it is conceivable that EGCG with its anti-oxidative properties also might be beneficial for the heart injured by global ischemia, which in case of local ischemia already has been demonstrated [20]. Therefore, the aim of our study was to evaluate in a model of Langendorff-perfused rabbit hearts subjected to cardioplegic arrest without and with EGCG, the impact of this catechin on myocardial function. Moreover, we also assessed the influence of EGCG on possible histological changes after ischemia and reperfusion.

## 2. Results

We evaluated 24 rabbits (weighting 1500–2000 g) with the following four experimental groups (*n* = 6 each): control (heart weight 7.65 ± 0.10 g), control + EGCG (heart weight 7.79 ± 0.13 g), cardioplegia (heart weight 7.63 ± 0.12 g), cardioplegia + EGCG (heart weight 7.61 ± 0.12 g). All groups did not differ significantly in heart weight.

### 2.1. Hemodynamic Parameter

At the end of the equilibration period, hemodynamic parameters were not significantly different between the 4 experimental groups.

**Figure 1:**
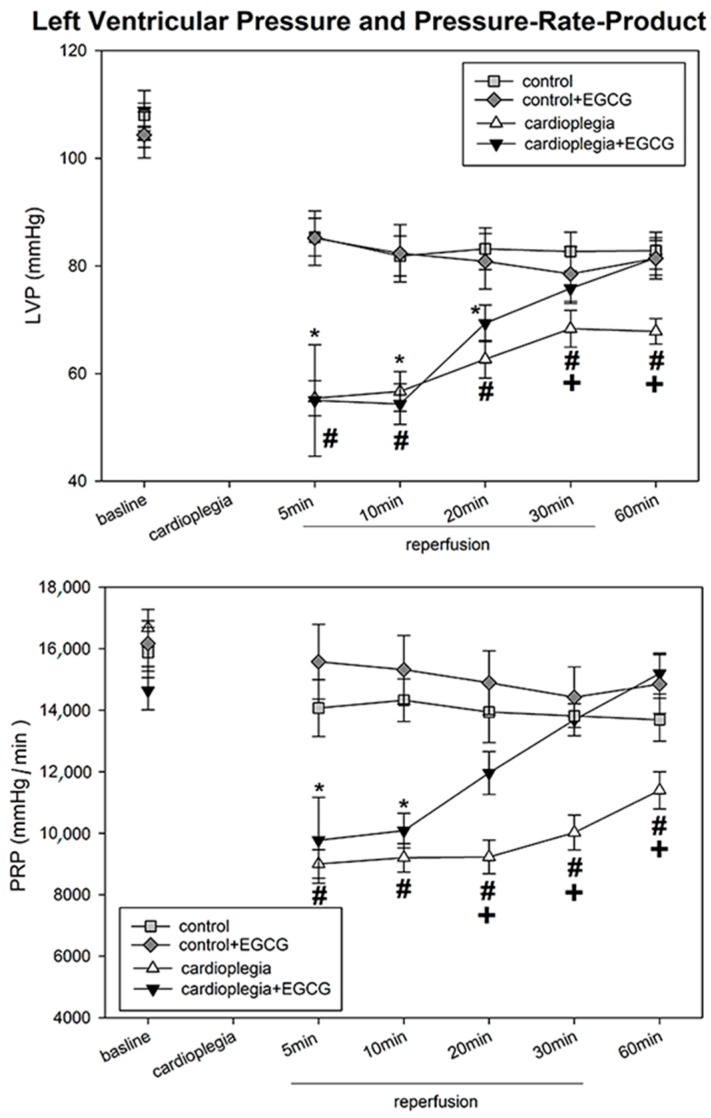
Upper Panel: Left ventricular pressure (LVP) in mmHg before (baseline) and during recovery from a 90 min period of cardioplegia. Cardioplegia was performed either without (white triangles, *n* = 6) or with addition of 20 µmol/L EGCG (black triangles, *n* = 6). Lower Panel: Pressure-rate-product (PRP) in mmHg/min before (baseline) and during recovery from a 90 min period of cardioplegia. Cardioplegia was performed either without (white triangles, *n* = 6) or with addition of 20 µmol/L EGCG (black triangles, *n* = 6). All data are given as means ± SEM. Significant differences (*p* < 0.05) between cardioplegia without EGCG versus control are indicated by **#**, significant differences between cardioplegia + EGCG versus control + EGCG by * and significant differences between cardioplegia without EGCG versus cardioplegia with EGCG by +.

**Figure 2:**
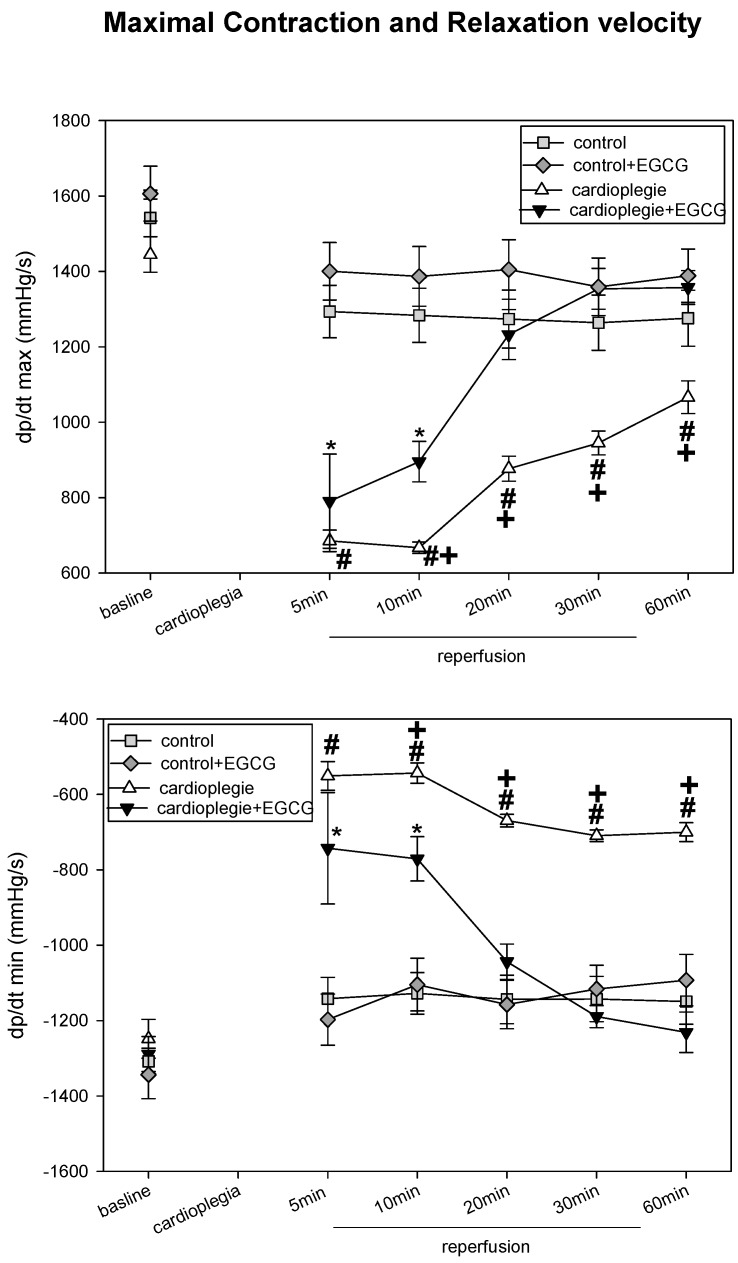
Upper Panel: Maximum contraction velocity (d*p*/d*t* max) in mmHg/s before (baseline) and during recovery from a 90 min period of cardioplegia. Cardioplegia was performed either without (white triangles, *n* = 6) or with addition of 20 µmol/L EGCG (black triangles, *n* = 6). Lower Panel: Maximum relaxation velocity (d*p*/d*t* min) in mmHg/s before (baseline) and during recovery from a 90 min period of cardioplegia. Cardioplegia was performed either without (white triangles, *n* = 6) or with addition of 20 µmol/L EGCG (black triangles, *n* = 6). All data are given as means ± SEM. Significant differences (*p* < 0.05) between cardioplegia without EGCG versus control are indicated by **#**, significant differences between cardioplegia + EGCG versus control + EGCG by * and significant differences between cardioplegia without EGCG versus cardioplegia with EGCG by +.

After 90 min cardioplegic arrest LVP (left ventricular pressure), PRP pressure-rate product, d*p*/d*t* max and min (maximal contraction and maximal relaxation velocity) were significantly impaired during the first 10 min of reperfusion compared to baseline levels (Figure 1 and Figure 2). However, the cardioplegic hearts recovered during the next 50 min. Application of EGCG during cardioplegia resulted in a significantly faster recovery to baseline levels. Interestingly, the ratio of CF/PRP (CF = coronary flow) was clearly elevated in both cardioplegia groups within the first 20 min, reaching control values after 30 min of reperfusion. The elevated CF/PRP-ratio indicated an O_2_-deficit and was not different in both cardioplegia groups (Table 1). The two-way ANOVA revealed that cardioplegia significantly altered the time course of LVP, d*p*/d*t* max and min (*p* < 0.05). Moreover, this effect was significantly affected by the presence of EGCG (*p* < 0.05) (=positive interaction of EGCG on time * cardioplegia).

In absence of cardioplegia EGCG alone had no effect.

In the cardioplegia group without EGCG, two out of six hearts, and in the cardioplegia + EGCG group, three out of six hearts, exhibited transient ventricular fibrillation (*p* > 0.05, not significant) at induction of reperfusion, which was mechanically terminated.

Analysis of electrophysiological data revealed that EGCG significantly reduced ARI in the control and also in the cardioplegia group during reperfusion (Table 1).

TAT was slightly prolonged during reperfusion in both groups, however, application of EGCG led to faster restoration (Table 1).

Vector similarity, BCL, HR, PQ-time, which reflects AV-node conduction time and QRS width were not significantly different between the four experimental groups (Table 1).

Arterio-venous pO_2_ difference was smaller in both cardioplegia groups, which means a decreased O_2_-extraction after cardioplegic arrest, indicating reduced oxygen consumption during the first 10 min of the reperfusion period parallel to reduced LVP.

ATP measurements revealed a significant decreased ATP levels in the cardioplegia group, which was normalized by EGCG application (Table 1).

In summary, it can be said that cardioplegic hearts treated with EGCG compared to cardioplegic hearts without EGCG had a significantly improved hemodynamic outcome with improved inotropic and lusitropic parameters.

The control hearts showed a slight deterioration in LVP and d*p*/d*t* max and min during the entire experimental time, without significant differences between the two control groups (without or with EGCG).

### 2.2. Histological Analysis

**Figure 3:**
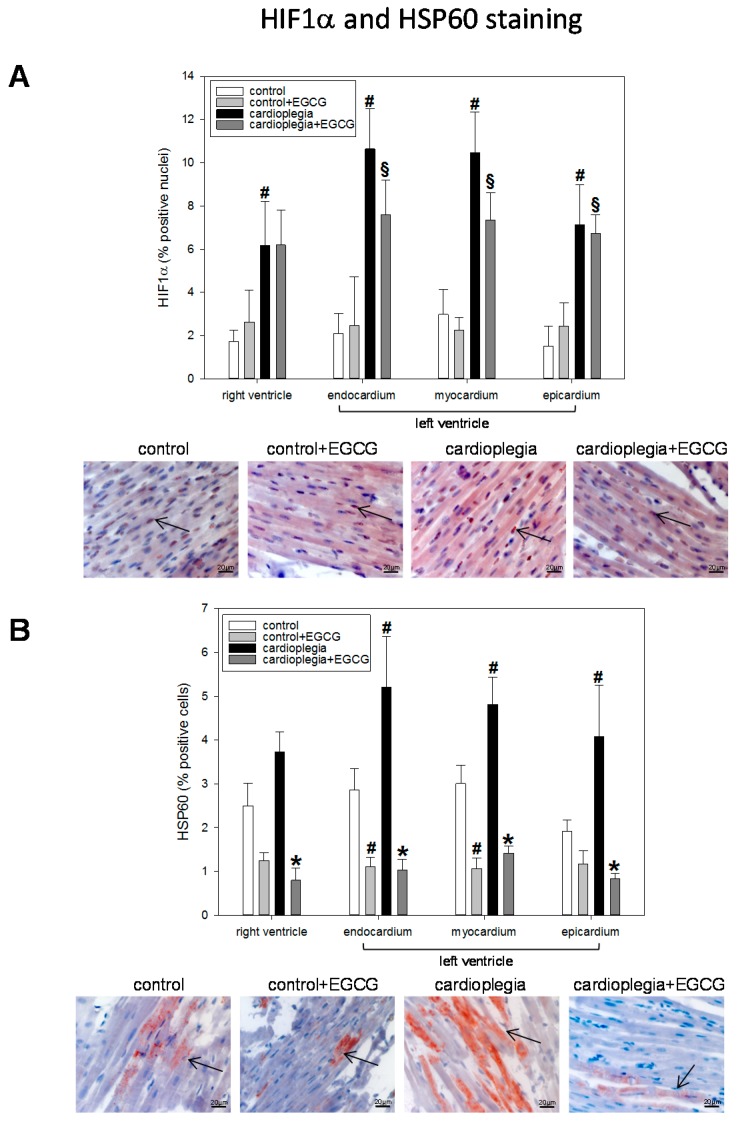
(**A**) Staining and quantification of HIF1α (hypoxia-inducible factor 1α) translocation. Bar graphs depict the percentage of nuclei positively stained for HIF1α in specimens from left ventricular epicardium, myocardium and endocardium and right ventricle after 90 min of cardioplegia followed by 60 min of recovery; (**B**) Staining and quantification of HSP60 (heat shock protein 60). Bar graphs depict the percentage of cells positively stained for HSP60 in specimens from left ventricular epicardium, myocardium and endocardium and right ventricle after 90 min of cardioplegia followed by 60 min of recovery. All data are given as means ± SEM. Significant differences (*p* < 0.05) versus control is indicated by **#**, significant differences versus control + EGCG by § and significant differences versus cardioplegia by *. Arrows indicate positively stained cells.

Histological analysis revealed a significant increase in HIF1α nuclear translocation in both cardioplegia groups and in all four heart regions assessed, indicating a significant ischemic burden of the heart during cardioplegia. Application of EGCG did not significantly influence the amount of HIF1α translocation (Figure 3A).

HSP60, a mitochondrial chaperonin, was also elevated during cardioplegia particularly in the left ventricular regions, and was significantly reduced by EGCG application. Interestingly, in the control hearts, EGCG also reduced HSP60 expression, at least in LV endocardium and myocardium (Figure 3B).

**Figure 4:**
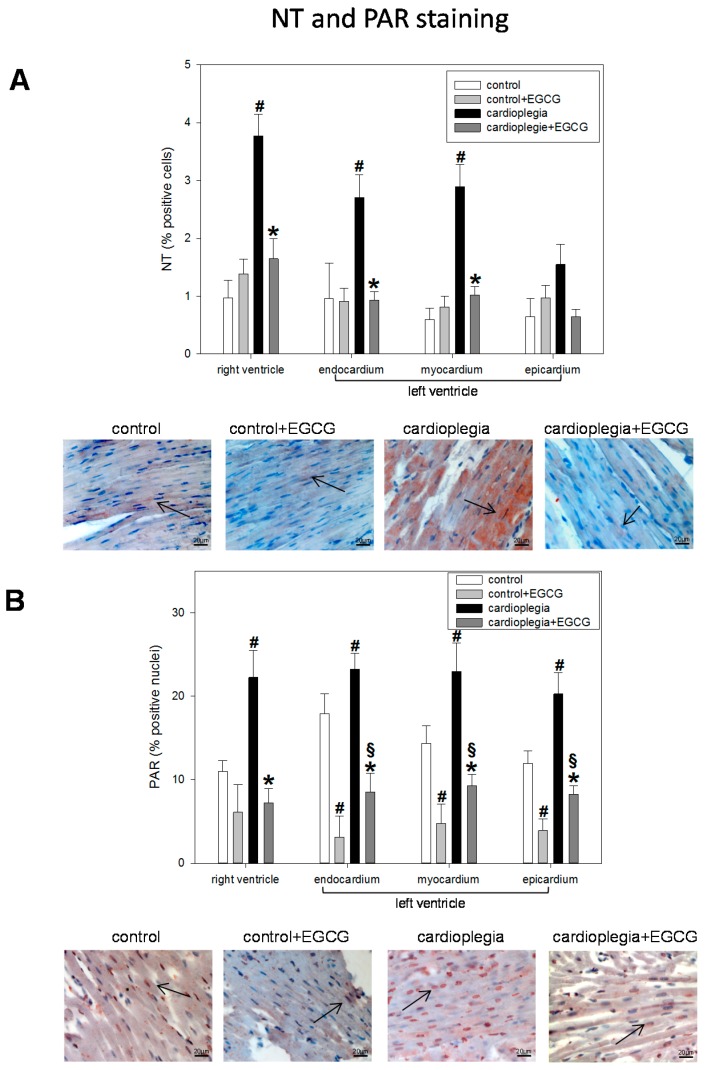
(**A**) Staining and quantification of NT (nitrotyrosine)-positive cells. Bar graphs depict the percentage of cells positively stained for NT in specimens from left ventricular epicardium, myocardium and endocardium and right ventricle after 90 min of cardioplegia followed by 60 min of recovery; (**B**) Staining and quantification of PAR (poly-ADP-ribose). Bar graphs depict the percentage of nuclei positively stained for PAR in specimens from left ventricular epicardium, myocardium and endocardium and right ventricle after 90 min of cardioplegia followed by 60 min of recovery. All data are given as means ± SEM. Significant differences (*p* < 0.05) versus control is indicated by **#**, significant differences versus control + EGCG by § and significant differences versus cardioplegia by *. Arrows indicate positively stained cells.

NT and PAR formation, a sign of nitrosative and oxidative stress, was significantly enhanced during cardioplegia. EGCG addition during cardioplegia significantly inhibited this increase. In case of PAR, ECGC also inhibited PAR formation under control conditions (Figure 4A,B).

**Figure 5:**
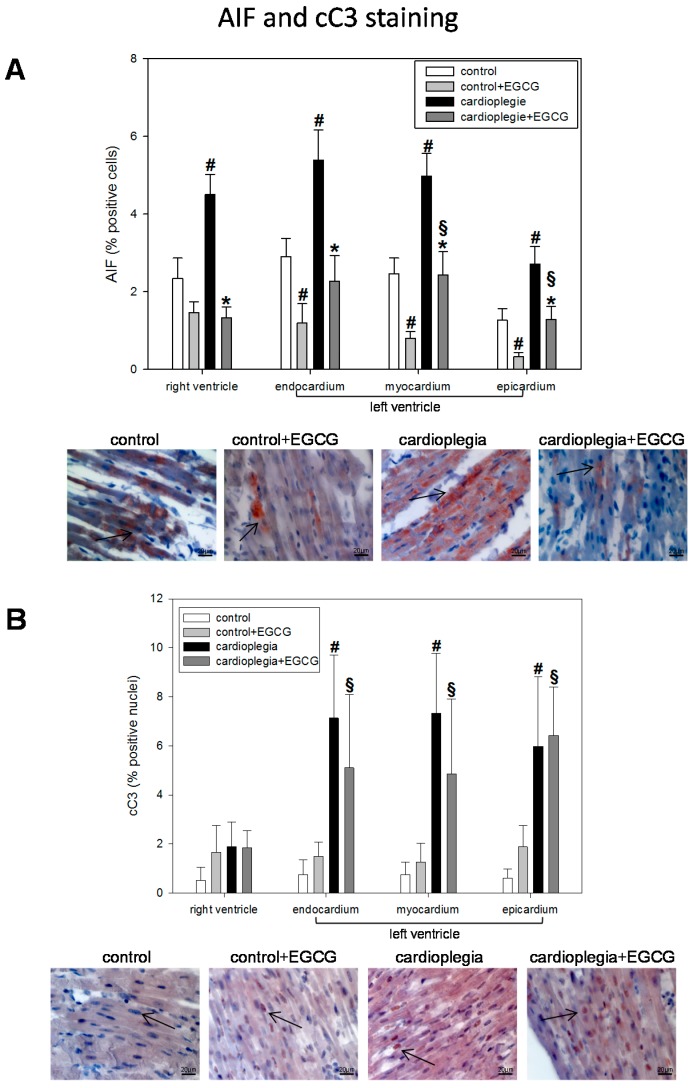
(**A**) Staining and quantification of AIF (apoptosis-inducing factor)-positive cells. Bar graphs depict the percentage of cells positively stained for AIF in specimens from left ventricular epicardium, myocardium and endocardium and right ventricle after 90 min of cardioplegia followed by 60 min of recovery; (**B**) Staining and quantification of cC3 (cleaved caspase 3). Bar graphs depict the percentage of nuclei positively stained for cC3 in specimens from left ventricular epicardium, myocardium and endocardium and right ventricle after 90 min of cardioplegia followed by 60 min of recovery. All data are given as means ± SEM. Significant differences (*p* < 0.05) versus control is indicated by **#**, significant differences versus control + EGCG by § and significant differences versus cardioplegia by *. Arrows indicate positively stained cells.

Moreover, cardioplegia significantly increased AIF nuclear translocation, which was significantly diminished by EGCG application during cardioplegia (Figure 5A). Additionally, in the three LV regions, EGCG also reduced AIF translocation in control hearts.

In contrast, EGCG had no influence on cardioplegia induced cC3 translocation: both cardioplegia groups exhibited significant cC3 translocation compared to control hearts (Figure 5B).

## 3. Discussion

During the entire experiment, control hearts (without or with EGCG) showed a slight decline in ventricular function. However, cardioplegic arrest which means global ischemia resulted in a severe deterioration of LVP, d*t*/d*t* max and min and PRP without complete recovery. These results might point to an irreversible impairment of left ventricular function and perhaps to a non-reversible injury of the heart.

EGCG application during cardioplegia led to a significant better outcome of the hearts, in the sense that inotropic and lusitropic parameters were significantly improved by EGCG. The cardioplegia + EGCG hearts reached control levels after 30 min of reperfusion, whereas the cardioplegic hearts without EGCG did not return to baseline levels during the 60 min of reperfusion.

Moreover, in the cardioplegia group markers of cellular damage were significantly enhanced and ATP-levels were significantly reduced compared to the control hearts. EGCG application during cardioplegia significantly lowered oxidative and nitrosative stress markers as well as AIF release. Furthermore, intracellular ATP-levels, which were decreased by cardioplegia could be restored by EGCG application.

It was proposed that EGCG application has a positive impact on the cardiovascular system: in animal studies using high-cholesterol-fed rats EGCG improved serum lipid profile, antioxidant enzymatic activities and reduced markers for myocardial damage [21]. Moreover, cardiac histopathological changes induced by high-cholesterol diet were abolished. Thus, even under non-ischemic conditions, EGCG showed beneficial effects on the cardiac muscle. Congruent with the results of Zhong et al., we also could demonstrate less AIF and HSP60 expression in the control + EGCG groups [21], indicating a more direct effect on cardiac cells. Thus, even without global ischemia EGCG seems to be useful for cellular metabolic processes.

In our study EGCG at a concentration of 20 µmol/L did not significantly affect cardiac hemodynamic parameters under control conditions (i.e., without cardioplegia), although a slight increase in d*p*/d*t* max and PRP was seen. Heart rate was slightly but not significantly elevated (and the corresponding BCL reduced) in both EGCG groups (without or with cardioplegia), a finding which corresponds well with the data of Bao et al. [22].

Interestingly, ARI was significantly reduced in both EGCG groups, which was not described before and which might be an intrinsic effect of EGCG. Moreover, as ARI reflects the action potential duration it might be that I_ca,L_ inhibiting properties of EGCG [23] may contribute to the shortening of ARI. However, in this case, one would expect an increase in CF/PRP ratio and an decrease in heart rate, which was not seen, so that this may be interpreted against an inhibiting effect on I_ca,L_.

TAT was elevated during reperfusion after cardioplegia which can be explained by the hyponatremic cardiac arrest using Custodiol^®^ (in this cardioplegic solution sodium concentration is 15mmol/L), which then leads to a depletion of cardiac sodium content with the result of a deceleration of excitation propagation. In the cardioplegia + EGCG group, this effect was less pronounced. The Na^+^/K^+^ imbalance is restored after reperfusion by the activity of the Na^+^/K^+^ ATPase under ATP consumption. One may argue that due to the ATP-preserving effect of EGCG (see below and Table 1) the function of these ATPases is improved.

The occurrence of ventricular fibrillation in both cardioplegia groups was not significantly different and other ECG parameters were also not influenced by EGCG in our experimental setup.

Because EGCG at higher concentrations (50 µmol/L) tends to generate rhythm disturbances, we used 20 µmol/L, which was sufficient to completely restore heart function after cardioplegia [22]. In a non-cardioplegic Langendorff-study (without administration of a cardioplegic solution) the positive EGCG effect on cardiac contraction and ATP-levels after global ischemic injury was also described, however, histological evaluation of the tissue and ECG measurements were not performed [24].

The question that now arises is how EGCG can improve ventricular contraction and relaxation. As it possesses anti-oxidative properties it might be that less reactive oxygen species occur during the reperfusion phase, which means less DNA-strand breaks, less mitochondrial damage, and less PARP (poly-ADP-ribose polymerase) activation (which we could demonstrate with reduced PAR formation and reduced nitrotyrosine- and HSP60-formation) [25]. This in turn means, a diminished ATP consumption by cellular repair processes, so that that more ATP is available for maintaining ion balances, ventricular contraction and relaxation, which is supported by the findings on TAT, LVP, d*p*/d*t* max and d*p*/d*t* min.

The ratio of CF/PRP was enhanced in both cardioplegia groups without significant differences. An increased CF/PRP ratio indicates an oxygen deficit which occurs during cardioplegia and which must be compensated during reperfusion. Although, EGCG was not beneficial in improving the oxygen deficit, it improved relaxation velocity and thus, decreased ventricular wall tension. As the heart is only perfused during diastole, a decreased wall tension should result in better hemodynamic outcome, which we also could demonstrate.

The arterio-venous partial oxygen pressure was decreased during the first 10 min of reperfusion in both cardioplegia groups. This can be explained by the fact that contractility of the cardioplegic hearts was severely reduced during this period and that some of the hearts exhibited ventricular fibrillation shortly after start of reperfusion with low oxygen extraction. After recovery of mechanical function, oxygen extraction rises to control levels.

Regarding our histological data, it is to say that HIF1α expression was not different between the two cardioplegia groups, indicating that the ischemic burden was similar among the groups. Production of reactive nitrogen species (RNS), which we assessed indirectly by the formation of the more stable nitrotyrosine product, was significantly enhanced by cardioplegia [26]. RNS might induce DNA strand breaks, which activate poly-ADP-ribose polymerase (PARP) leading to enhanced PAR formation, which we also demonstrated. PARP activation is an important process regarding cell survival as DNA damage is repaired but is also an ATP-consuming process [27]. EGCG with its radical scavenging properties effectively reduced NT- and also PAR-formation, indicating less activation of PARP, which might have contributed to the restored ATP-levels in the cardioplegia + EGCG group [28]. Additionally, the reduced level of HSP60 in both EGCG groups (during cardioplegia and also during control conditions) might point to a cell-protective effect of EGCG. Moreover, apoptosis-inducing factors (AIF and cC3) were enhanced after global ischemia (although the number of cells being positive for AIF and cC3 was still low after cardioplegia). While EGCG reduced AIF expression, it did not reduce cC3 nuclear translocation. Interestingly, at least in neuronal cells, polyphenols inhibit AIF release from mitochondria [29]. CC3 effects, however, did not reach significance between cardioplegia and cardioplegia + EGCG. This may be due to the fact that cC3 activation is the last step in the pathway of hypoxia, free radicals, DNA strand breaks, PAR-formation/AIF-translocation and apoptosis induction. Thus, in our study, cC3 started to increase involving about 7% of the nuclei, but probably the final level of activation will not be reached within the time scale of our Langendorff setup (i.e., 90 min ischemia and 60 min reperfusion), so that differences in cC3 will probably take several hours, which cannot be investigated in this type of model [30].

## 4. Materials and Methods

All experiments were performed in accordance with the ethical rules of the Council for International Organization of Medical Science and the German/European laws for animal welfare. The study was approved by our institutional ethical committee for animal welfare and the regional council of Leipzig named “Landesdirektion Sachsen” (reference number T21/16). The investigation conforms to the Directive 2010/63/EU of the European Parliament as well as to the Guide for the Care and Use of Laboratory Animals published by the US National Institutes of Health (NIH Publication No. 85-23, revised 1996).

Twenty-four Chinchilla Bastard rabbits (body weight: 1500–2000 g) were allocated to the following groups (each *n* = 6): control, control + EGCG 20 µmol/L, cardioplegia, cardioplegia + EGCG 20 µmol/L. Preparation of the hearts and perfusion according to the Langendorff-technique was performed according to [31]. Briefly, rabbits were anaesthetized with an intramuscular injection of medetomidine (0.2 mg/kg body weight) and ketamine (20 mg/kg body weight). To prevent from clotting we administered heparin (500 IU/kg body weight) intravenously. After induction of narcosis the rabbits were exsanguinated by opening of the carotid arteries. Subsequently, the thorax was opened and aorta and pulmonary artery were cannulated. The caval and pulmonary veins were ligated and the hearts were quickly removed and connected to the Langendorff apparatus. Via the aortic cannula the coronary arteries were perfused with Tyrode’s solution (containing (in mmol/L) Na^+^ 161, K^+^ 5.36; Ca^2+^ 1.8, Mg^2+^ 1.05; Cl^−^ 148, HCO_3_^−^ 23.8, PO_4_^3−^ 0.42 and glucose 11.1; gassed with 5% CO_2_ and 95% O_2_), at a constant pressure of 75 cmH_2_O. Coronary flow (CF mL/min) was measured via the pulmonary artery. A balloon was inserted via the left atrium into the left ventricle, filled with water and an end-diastolic pressure of 8.8 ± 0.5 mmHg was established. The balloon was connected to a pressure transducer and a two-channel bridge amplifier (Hugo Sachs Elektronik, March-Hugstetten, Germany). Pressure and its first derivative traces were continuously recorded (trace recorder: Recomed, Hellige, Freiburg, Germany) to determine left ventricular developed systolic pressure (LVP) [mmHg], d*p*/d*t* max (i.e., maximal contraction velocity) (mmHg/s) and d*p*/d*t* min (i.e., maximal relaxation velocity) [mmHg/s], basic cycle length (BCL) [ms] and heart rate (HR) [min^−1^]. From these we calculated the pressure-rate-product (PRP) as LVP × HR (mmHg/min). In order to assess coronary supply in relation to the workload performed by the heart we also calculated the relative coronary flow as CF/PRP (µL/mmHg). The hearts were spontaneously beating.

Four polyester plates with 64 electrodes each (i.e., 256 AgCl electrodes) were attached to the surface of the heart in an elastic manner so that they could follow the hearts movements easily without dislocation. The 4 × 64 electrodes were connected to a 254 channel mapping system HAL4 (temporal resolution: 4 kHz/channel; amplitude resolution: 0.04 mV) to measure surface ECGs from the right and left ventricular free wall and from the anterior and posterior wall. We determined the time of activation at each electrode as the time of the fastest negative intrinsic deflection t(d*U/*d*t* min) [32]. The repolarization time was determined as t(d*U/*d*t* max) during T-wave according to [32]. From these data the activation-recovery interval (ARI) [ms] was calculated for each electrode representing the epicardial potential duration. Moreover, the total activation time (TAT) [ms] as the delay between the earliest and the latest activation time was determined. In addition, PQ-interval (atrio-ventricular conduction time) and QRS width were assessed according to [32]. From the activation data of a given electrode and it neighbors it was possible to calculate a vector giving direction and velocity of the activation wave [32]. By comparing vectors of two heart beats it was possible to define the percentage of vectors with unchanged direction (unchanged = less than 5° deflection) for each electrode giving the vector field similarity [32].

Additionally, via the pulmonary cannula pO_2_ (partial oxygen pressure in mmHg), pCO_2_ (partial carbon dioxide pressure in mmHg) and coronary flow (measured volumetrically) (mL/min) were assessed during the entire experiment. Oxygen partial pressure was measured in Tyrode’s solution (“arterial”) and in the outflow of pulmonary cannula (“venous”) and the “arterio-venous” difference, which is a measure for the oxygen extraction of the beating heart, was calculated.

### 4.1. Experimental Protocol

Rabbits were randomly assigned to either the control group or to the cardioplegia group without or with EGCG, respectively.

In one series of experiments control hearts were evaluated over the entire test period to assess changes in hemodynamic or electrophysiological parameters (=control). Moreover, to test whether EGCG has an intrinsic effect on cardiac hemodynamics or on ECG parameters, EGCG (Sigma-Aldrich, Taufkirchen, Germany) was dissolved in water and added to the Tyrode’s solution to achieve a final concentration of 20 µmol/L (=control + EGCG). 

For the cardioplegia experiments the hearts were arrested using the cardioplegic solution Custodiol^®^ (Dr. Franz Köhler Chemie, Alsbach-Hähnlein, Germany; containing (in mmol/L): NaCl 15, KCl 9, MgCl_2_ 4, CaCl_2_ 0.015, histidinehydrochloride 18, l-histidine 180, tryptophan 2, mannitol 30 and ketoglutarate/glutamic acid 1) (=cardioplegia). In further experiments, to evaluate possible effects of EGCG on cardioplegic arrest, Custodiol^®^ was supplemented with 20 µmol/L EGCG (=cardioplegia + EGCG). 

At the end of the equilibration period of 45 min baseline hemodynamic parameters and epicardial electrograms were recorded as well as arterial and venous pO_2_ and pCO_2_.

To investigate the impact of EGCG on cardioplegic arrest, Langendorff-perfusion was stopped and 30 mL Custodiol^®^ was injected in the coronary arteries without or with EGCG addition and the hearts stayed arrested for 90 min. The control hearts, spontaneously beating, did not receive Custodiol^®^, instead EGCG in the same concentration was added to the Tyrode’s solution. After 90 min cardioplegic arrest reperfusion with Tyrode’s solution was initiated and the above described parameters were measured at 5, 10, 20, 30 and 60 min. The control hearts, which were perfused with Tyrode’s solution the whole period, were also evaluated at the specified time points.

Thereafter, the hearts were quickly removed from the Langendorff-apparatus and samples from the left ventricular apex were shock frozen in liquid nitrogen for ATP analysis (HPLC). For histological investigations transverse two-chamber slices of the heart were fixed in 4% neutral buffered formalin and embedded in paraffin.

### 4.2. Histology

Histological evaluation of the ventricular specimen was carried out according to [33]. To analyze HIF1α (hypoxia induced factor 1α)-translocation, HSP60 (heat shock protein 60)-levels, nitrotyrosine and PAR (poly-ADP-ribose) formation as well as the pro-apoptotic factors AIF (apoptosis inducing factor) and cC3 (cleaved caspase 3), 2 µm slices were cut, de-paraffinized, re-hydrated and either cooked in 0.01 mol/L citrate buffer (pH = 6) (for AIF-, HIF1α-, PAR-, cC3- and HSP60-staining) or treated with 0.3% Triton-X 100 (for nitrotyrosine-staining).

After blocking unspecific binding with bovine serum albumin (BSA), specimens were incubated with either mouse monoclonal anti-nitrotyrosine primary antibody (1:50, Merck-Millipore, Darmstadt, Germany), mouse monoclonal anti-PAR antibody (1:600, Bio-Rad, Munich, Germany), mouse anti-AIF primary antibody (1:50, Santa Cruz, Heidelberg, Germany), with mouse monoclonal anti-HIF1α primary antibody (1:50, Thermo Scientific, Dreieich, Germany), with mouse monoclonal HSP60 primary antibody (1:250, Abcam, Cambridge, UK) or with rabbit anti-cC3 primary antibody (1:300, New England Biolabs, Frankfurt, Germany) over-night at 4 °C. 

Thereafter, specimens were washed three times and signal detection was carried out with the appropriate secondary HRP-labelled antibodies (horseradish-labelled goat anti-mouse or goat anti-rabbit secondary antibodies 1:200, Sigma-Aldrich, Taufkirchen, Germany) and the red chromogen AEC (3-amino-9-ethylcarbazole, Dako, Hamburg, Germany) according to the manufacturer’s instruction. Nuclei of cardiomyocytes were counter-stained with haemalum.

All slides were investigated at 400× magnification using a Zeiss Axiolab microscope (Zeiss, Jena, Germany) and photographs were randomly taken by a blinded investigator. We analyzed the mid-myocardial region of the right ventricular free wall (RV), and the epicardium, mid-myocardium and endocardium of the left ventricle (LV).

Each region was evaluated separately. At least 50 cells per region were investigated and the ratio of positive cells (red nuclei or red cytoplasm) was calculated in relation to the total number of counted cells. Accordingly, 200 cells per heart and 1200 cells per experimental group were examined.

#### HPLC Analysis of ATP Content

Left ventricular apices were homogenized on ice with 0.4 mmol/L perchloric acid and precipitated with KOH. The samples were centrifuged for 10 min (3000× *g*) and the supernatants were injected twice onto a pre-equilibrated RP18 column (LiChroCART, Merck, Darmstadt, Germany) as previously published [31]. For ATP detection a HPLC-apparatus from Knauer (Berlin, Germany) and an UV-detector (PDA Detector 2800, Knauer, Berlin, Germany) were used. Peaks were measured at 259 nm. Standard curves were generated with 4 concentrations of ATP, (25-12.5-6.25-3.125 µg/mL). External standards and ventricular samples were measured together in one HPLC run.

### 4.3. Statistical Analysis

All results are presented as mean value and standard error of mean (SEM) of *n* = 6 experiments. Normal distribution of the data was tested with Shapiro-Wilk’s test.

Hemodynamic data were evaluated with two-way ANOVA (cardioplegia*EGCG; time*EGCG) followed by Students *t*-test with Bonferroni correction for multiple measurements to detect statistical significance at a level of *p* < 0.05. ATP measurements were tested for statistical significance using ANOVA followed by Students *t*-test, and the non-parametric Kruskal-Wallis test was performed to analyse the histological data (*p* < 0.05).

Prevalence of ventricular fibrillation in both experimental groups was tested using Fischer’s exact test.

For the statistical analysis Systat for Windows, version 13 (Systat Inc., Evanston, IL, USA) was used.

## 5. Conclusions

Our data indicate that EGCG improves functional recovery of the heart after cardioplegic arrest probably due to its anti-oxidative, radical-scavenging and PARP-inhibiting effects, which help to preserve intracellular ATP. EGCG, thus, may be an interesting additive for cardioplegic solutions worth further investigations.

## Figures and Tables

**Table 1:** Physiological, biochemical and hemodynamic parameters.

Parameters	Baseline	Reperfusion 5 min	Reperfusion 10 min	Reperfusion 20 min	Reperfusion 30 min	Reperfusion 60 min
ARI (ms) control control + EGCG cardioplegia cardioplegia + EGCG	126 ± 3.9 133 ± 4.4 138 ± 4.7 131 ± 3.5	136 ± 3.5 126 ± 4.9 150 ± 6.5 141 ± 3.7 §	139 ± 4.3 124 ± 4.6 # 150 ± 4.5 136 ± 4.2 §,*	142 ± 4.5 121 ± 4.3 # 148 ± 5.6 132 ± 4.4 #,*	140 ± 3.8 122 ± 4.5 # 145 ± 5.7 126 ± 3.2 *	137 ± 3.6 123 ± 4.6 # 141 ± 5.6 125 ± 2.9 *
TAT (ms) control control + EGCG cardioplegia cardioplegia + EGCG	14.3 ± 1.8 13.7 ± 1.0 15.1 ± 1.6 14.9 ± 1.2	13.9 ± 2.6 15.0 ± 0.9 18.0 ± 2.1 18.3 ± 1.2	15.6 ± 3.5 13.9 ± 0.9 19.3 ± 2.3 19.8 ± 2.5	13.4 ± 1.1 15.6 ± 1.1 18.4 ± 1.6 17.0 ± 2.2	13.1 ± 0.5 14.7 ± 0.6 20.0 ± 2.0 # 17.0 ± 2.2	13.4 ± 1.4 14.4 ± 0.9 19.9 ± 2.2 # 16.5 ± 2.1
Vector similarity (%) control control + EGCG cardioplegia cardioplegia + EGCG	55 ± 7.3 58 ± 7.9 54 ± 5.5 58 ± 7.6	35 ± 9.5 33 ± 9.4 22 ± 5.3 17 ± 5.0	30 ± 8.3 30 ± 8.6 22 ± 5.5 20 ± 5.8	36 ± 9.2 33 ± 8.2 25 ± 6.2 21 ± 4.4	34 ± 8.9 32 ± 8.8 24 ± 6.9 23 ± 4.9	33 ± 7.9 30 ± 9.9 23 ± 6.2 24 ± 4.9
PQ (ms) control control + EGCG cardioplegia cardioplegia + EGCG	64 ± 2.0 62 ± 1.3 63 ± 2.0 64 ± 1.5	64 ± 0.9 61 ± 1.6 65 ± 2.9 68 ± 4.0	64 ± 1.0 60 ± 1.2 64 ± 3.6 60 ± 2.4	64 ± 1.1 65 ± 1.7 64 ± 2.5 66 ± 1.3	65 ± 1.2 62 ± 2.5 65 ± 2.1 66 ± 1.3	64 ± 1.4 63 ± 2.6 63 ± 2.4 63 ± 0.7
QRS (ms) control control + EGCG cardioplegia cardioplegia + EGCG	28 ± 1.0 25 ± 0.6 26 ± 0.6 24 ± 0.7	27 ± 1.0 27 ± 0.6 25 ± 0.4 27 ± 1.2	28 ± 0.8 25 ± 0.7 25 ± 0.7 28 ± 1.0	26 ± 0.7 27 ± 0.7 26 ± 0.4 26 ± 0.6	27 ± 1.0 26 ± 0.6 24 ± 0.4 24 ± 0.8	28 ± 0.8 27 ± 0.5 25 ± 0.9 26 ± 1.0
BCL (ms) control control + EGCG cardioplegia cardioplegia + EGCG	375 ± 12 359 ± 8 387 ± 10 366 ± 9	390 ± 20 352 ± 19 365 ± 9 327 ± 21	400 ± 21 332 ± 14 368 ± 13 322 ± 10	399 ± 22 339 ± 16 421 ± 16 356 ± 8	393 ± 24 337 ± 15 434 ± 24 338 ± 10	400 ± 25 350 ± 20 375 ± 15 328 ± 9
HR (min^−1^) control control + EGCG cardioplegia cardioplegia + EGCG	163 ± 5.3 170 ± 3.6 159 ± 4.0 166 ± 4.2	161 ± 6.7 182 ± 7.5 167 ± 4.7 188 ± 11	157 ± 6.9 188 ± 6.1 170 ± 6.5 192 ± 6.5	158 ± 6.8 187 ± 7.4 150 ± 7.0 171 ± 4.2	162 ± 7.7 187 ± 7.2 150 ± 7.6 182 ± 5.4	160 ± 7.9 185 ± 8.4 168 ± 6.7 187 ± 5.1
CF/PRP (µL/mmHg) control control + EGCG cardioplegia cardioplegia + EGCG	1.86 ± 0.12 1.71 ± 0.06 1.81 ± 0.04 1.68 ± 0.06	1.83 ± 0.06 1.74 ± 0.09 3.09 ± 0.11 # 3.33 ± 0.47 §	1.73 ± 0.05 1.75 ± 0.09 2.78 ± 0.14 # 2.95 ± 0.16 §	1.88 ± 0.06 1.78 ± 0.06 2.35 ± 0.08 # 2.27 ± 0.12 §	1.90 ± 0.07 1.80 ± 0.05 2.05 ± 0.04 1.96 ± 0.08	1.74 ± 0.05 1.77 ± 0.09 1.91 ± 0.10 1.89 ± 0.09
pO_2_art-ven (mmHg) control control + EGCG cardioplegia cardioplegia + EGCG	400 ± 11 398 ± 11 384 ± 8.0 404 ± 6.7	411 ± 6.5 401 ± 9.4 313 ± 10 # 262 ± 6.2 §	400 ± 6.3 407 ± 11 312 ± 9.0 # 285 ± 7.2 §	414 ± 5.7 406 ± 11 360 ± 6.9 368 ± 8.7	425 ± 5.3 411 ± 13 360 ± 9.0 387 ± 7.7	435 ± 6.0 433 ± 12 368 ± 8.6 380 ± 8.8
ATP content µg/g control control + EGCG cardioplegia cardioplegia + EGCG						60 ± 6.5 73 ± 9.0 43 ± 8.2 # 69 ± 5.4 *

ARI activation recovery interval, TAT total activation time, PQ PQ interval, QRS QRS complex, BCL basic cycle length, HR heart rate, CF coronary flow, PRP pressure rate product, pO_2_art-ven difference of arterio-venous partial oxygen pressure, **#** significant differences versus control; * significant differences versus cardioplegia; § significant differences versus control + EGCG, (*p* < 0.05).

## Abbreviations

AIF	Apoptosis inducing factor
ARI	Activation recovery interval
ATP	Adenosine triphosphate
BCL	Basic cycle length
cC3	Cleaved caspase 3
CF	Coronary flow
d*p*/d*t* max	Maximal contraction velocity
d*p*/d*t* min	Maximal relaxation velocity
ECG	Electrocardiogram
EGCG	(−)-Epigallocatechin gallate
HIF1α	Hypoxia induced factor 1α
HPLC	High pressure liquid chromatography
HR	Heart rate
HSP60	Heat shock protein 60
Ica,L	l-type calcium channel
LV	Left ventricle
LVP	Left ventricular pressure
NT	Nitrotyrosine
PAR	Poly-ADP-ribose
PARP	Poly-ADP-ribose polymerase
pO_2_	Partial oxygen pressure
PQ	PQ-interval
PRP	Pressure-rate product
QRS	QRS-interval
RNS	reactive nitrogen species
RV	Right ventricle
TAT	Total activation time
